# Recent Advances and Future Perspectives in Microbial Phototrophy in Antarctic Sea Ice

**DOI:** 10.3390/biology1030542

**Published:** 2012-10-22

**Authors:** Eileen Y. Koh, Andrew R. Martin, Andrew McMinn, Ken G. Ryan

**Affiliations:** 1 School of Biological Sciences, Victoria University of Wellington, PO Box 600, Wellington 6140, New Zealand; Email: mickye@nus.edu.sg; 2 Institute for Marine and Antarctic Studies, University of Tasmania, Hobart 7001, Australia; Email: andrew.martin@utas.edu.au (A.R.M.); andrew.mcminn@utas.edu.au (A.M.)

**Keywords:** phototrophic bacteria, cyanobacteria, aerobic anoxygenic phototrophic bacteria, proteorhodopsin, Antarctic sea ice

## Abstract

Bacteria that utilize sunlight to supplement metabolic activity are now being described in a range of ecosystems. While it is likely that phototrophy provides an important competitive advantage, the contribution that these microorganisms make to the bioenergetics of polar marine ecosystems is unknown. In this minireview, we discuss recent advances in our understanding of phototrophic bacteria and highlight the need for future research.

## 1. Introduction

Microorganisms have been fundamentally important to the history and function of life on Earth. They have played a central role in the climatic, geological, and biological evolution of the planet [1]. They are found in every conceivable ecological niche, from the tropics to the poles, from underground mines and oil fields to the stratosphere and mountain ranges, from deserts to the Dead Sea and from hot springs to underwater hydrothermal vents [2,3,4,5]. Microbes dominate the flux of energy and biologically important chemical elements in the world’s oceans and, as a result, are estimated to be five to ten times the mass of all multicellular marine organisms [6]. Bacteria harbor a potential reservoir of useful genes for medicine and biotechnology, and unraveling their complex taxonomic diversity is considered the key to understanding the process of evolution [7,8].

**Figure 1:**
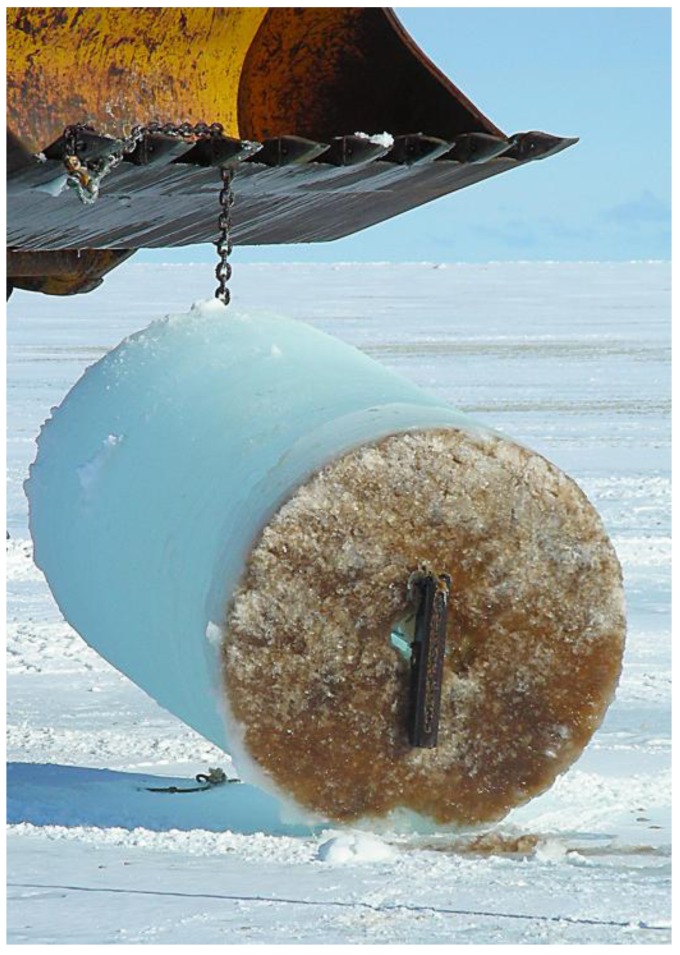
Cross-section of sea ice. A distinct brown coloration is present at the bottom 20 cm of a 1 m diameter section of sea ice. This is due to the high concentration of bacteria and microalgae within the sea ice.

Sea ice is one of the most seasonally dynamic ecosystems on Earth. An important driver of the global climate system, annual sea ice at polar latitudes influences both physical and biological processes; particularly in modulating the exchange of heat and moisture between the atmosphere and ocean, and restricting the penetration of solar radiation. Importantly, sea ice also provides a stable platform for the colonization and growth of marine microbes [9,10]. Although a range of microbial taxa are initially scavenged from the water column during ice formation, only some are able to adapt to the physicochemical variability that characterizes the brine inclusions and interstices of the ice matrix. The most conspicuous ice-bound organisms are microalgae and research efforts have historically focused on the composition, physiology, and ecology of the diatoms that dominate sea ice assemblages [11,12,13,14,15]. Sea ice algae contribute between 10%–28% of the total primary production in ice-covered regions of the Southern Ocean [10,16] and over 90% of this biogenic carbon is produced within first-year ice and approximately 60% during the austral spring (November-December) when the algal cells typically discolor the bottom 1–20 cm of the ice [16] (Figure 1). Microalgae provide a crucial source of winter nutrition for juvenile zooplankton such as the Antarctic krill *Euphausia superba* [17,18], and may provide inocula for bloom events at the receding ice edge in the austral summer [11,16,19,20].

While bacteria are now recognized as a major biological component of the oceanic carbon cycle and ecosystem structure [21,22], an understanding of the phylogenetic diversity and functional capabilities of ice-associated bacteria remains fragmentary [23]. Evidence that bacteria actively grow within the ice dates back to only the 1980’s when Sullivan and Palmisano [24] observed large and morphologically distinct bacteria undergoing cell division in fast-ice within McMurdo Sound, Antarctica. This initial observation indicated an active heterotrophic community, and the subsequent microautoradiographic uptake of radiolabeled compounds such as ^14^C-L-serine, ^3^H-serine, ^3^H-glucose and ^3^H-thymidine confirmed community-level activity in the form of DNA synthesis [24,25]. More recent single-cell analyses, including the use of tetrazolium chloride (CTC) and fluorescence *in situ* hybridization (FISH), have shown that ~80% of the bacteria present in the bottom of Antarctic sea ice have a probe-positive cellular rRNA content and >30% of the cells have an electron transport system that is capable of reducing CTC [26,27]. Most of these cells appear to be heterotrophic bacteria, which either live freely or attached to microalgae or detritus [28,29]. Molecular-based surveys of SSU rRNA gene diversity typically reveal psychrophilic and halotolerant members of the Proteobacteria, Bacteroidetes (previously known as the Cytophaga-Flavobacteria-Bacteroides (CFB) cluster) and Gram-positive bacteria [23,28,30].

**Figure 2:**
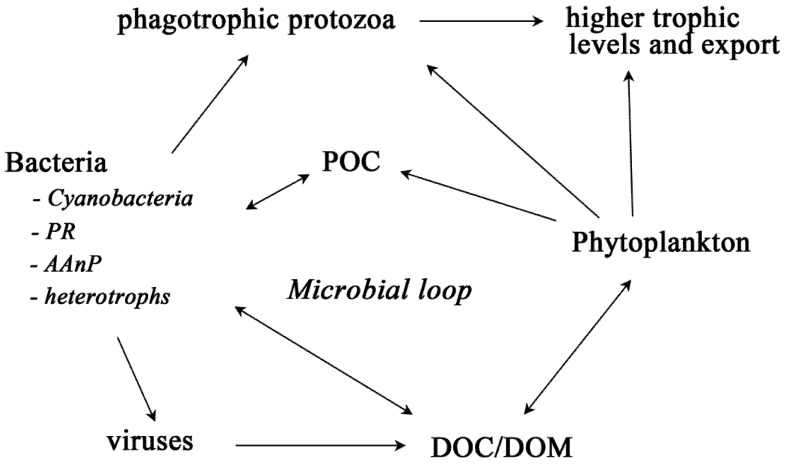
Sea ice food web and the microbial loop. The microbial loop re-drawn and abridged from Azam *et al.* (1983) and Fenchel (2008). Only the bacteria discussed in this review are presented; the other bacteria are grouped as heterotrophs. AAnP = aerobic anaerobic phototroph, DOC = dissolved organic carbon, DOM = dissolved organic matter, POC = particulate organic carbon, PR = proteorhodopsins.

Following a decade of seminal research conducted within McMurdo Sound, Antarctica, Sullivan [25] suggested that sea ice bacteria might play an important role in secondary microbial production mediated through the microbial loop and remineralisation and recycling of ice-associated organic matter (Figure 2). Sullivan [25] also postulated that these bacteria maintain a balance of oxygen concentration in the ice microenvironment through their respiration and may be involved in ice nucleation and early stages of sea ice formation although these ideas remain largely unsubstantiated.

## 2. Bacteria with Light-Harvesting Capabilities

The energy to support life in the sea is ultimately derived from phototrophy in the euphotic zone [31]. The most significant contribution from prokaryotic life forms is from cyanobacteria, which utilize chlorophyll-based phototrophy and contribute 30% of all globally fixed carbon [32]. However, in recent years, non-cultivation-based studies of bacteria have led to the discovery of novel genes, proteins and phototrophic mechanisms that are rapidly gaining scientific interest [33,34,35,36,37]. In particular, widespread reports of bacteriochlorophyll (bchl) and proteorhodopsin (PR) in planktonic marine prokaryotes are challenging the assumption that chl-*a* is the only light-capturing pigment of ecological importance. It remains to be seen whether these metabolic pathways will require a significant revision of oceanic energy budgets [22,38], but alternative light-based metabolic strategies are now being described in aquatic ecosystems that range from the deep-sea biosphere to high-altitude glaciers [39,40].

### 2.1. Cyanobacteria

Cyanobacteria colonize a variety of polar terrestrial ecospheres including rocks, glaciers, ice shelves, streams, ponds and lakes [41,42,43,44,45]. Phototrophs in these environments are generally psychrotolerant and exhibit an assortment of cold-protection mechanisms and slow growth rates to endure freeze-thaw cycles. The intracellular accumulation of salts to sustain osmotic balance, variation in DNA repair mechanisms and the use of photo-complexes are additional strategies that terrestrial cyanobacteria employ in extreme cold environments [44,46,47]. Picocyanobacteria such as *Synechococcus* and *Prochlorococcus* are the most abundant phototropic cells in the World’s oceans [48]. Despite this significant contribution to primary production, these cells are small, 0.5–1.5 µm and, in the case of *Prochlorococcus*, remained undetected until 1986 when they were discovered using flow cytometry [49]. Importantly, the abundance of marine cyanobacteria decreases rapidly south of latitude 40° [50,51,52] and this has been attributed to eco-physiological factors such as temperature, salinity and nutrient requirements [44].

Considering their prevalence in cold terrestrial environments, the apparent absence of cyanobacteria within sea ice is, however, unexpected. Interestingly, *Synechococcus* was detected from coastal waters off East Antarctica in 1989 using microscopy and pigment chemistry [53] and a decade later, cyanobacterial-like pigments (phycoerythrin and phycocyanin) were detected for the first time within the ice matrix using flow cytometry [54]. Pigment-based confirmation is questionable however as phycoerythrin and phycocyanin are also present in other algae including Cryptophytes which are common during Antarctic coastal blooms [54]. To potentially validate these earlier findings, a multi-method molecular analysis was recently carried out on fast-ice cores extracted from sites spanning 300 km in the Ross Sea region of Antarctica [55]. Clone libraries were constructed from the 16S rDNA gene, the internal transcribed sequence (ITS) region and the cyanobacterial core RNA polymerase (*rpo*C). Analysis of all sections of extracted ice did not reveal the presence of *Synechococcus* sp.*, Prochlorococcus* sp., or any other marine cyanobacteria-related species. Additional screening was carried out using ligation detection reaction-based microarray, which can detect as little as 1 *f*mol of DNA [56]. Data from the ITS and microarray analysis of these sea ice samples showed close affiliation to the freshwater cyanobacteria *Phormidium* sp. and *Cylindrospermopsis* sp., respectively [55], but the closest Antarctic relative was an uncultured cyanobacteria clone from the nearby meromictic Lake Fryxell [42]. Aerobiology studies conducted in the Antarctic [57,58] and the Arctic [59] suggested that much of the biological material present in the air originates locally. Harding *et al.* [59] observed that >47% of the operational taxonomic units (OTUs) in Arctic snow samples were from previously reported local cyanobacteria [45,60]. Given that Terra Nova Bay is situated in a katabatic wind cross-zone [61], it is likely that the cyanobacterial propagules identified by Koh *et al*. [55] were wind-transported from nearby freshwater ponds or terrestrial soil and incorporated into the ice during seasonal formation. Despite earlier anecdotal findings, molecular-based evidence now confirms that cyanobacteria do not play a significant role in sea ice ecosystem dynamics.

### 2.2. Bacteriochlorophyll

**Figure 3:**
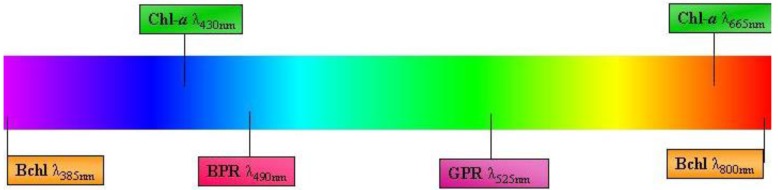
Schematic diagram of light pigments/proteins of sea ice phototrophic bacteria. Bchl = bacteriochlorophyll; Chl-*a* = Chlorophyll-*a*; BPR = Blue proteorhodopsin; GPR = Green proteorhodopsin. Diagram not drawn to scale.

Phototropic metabolism is a feature of four other eubacterial phyla (*i.e.*, Proteobacteria, Chlorobi, Chloroflexi and Firmicutes). Unlike cyanobacteria, these phototrophs utilize the most ancient form of photosynthesis: anoxygenic photosynthesis [62]. This pathway is important for nitrogen-fixation, and cells with bacteriochlorophyll (bchl) also play an important role in the microbial loop [63,64]. The presence of highly diverse anoxygenic phototrophic bacterial communities in marine environments now suggests that non-chlorophyll-*a* phototrophy may be a more common life history strategy than previously realized. For example, the Proteobacteria contain the largest group of anoxyphototrophs [65], which were thought to be strictly anaerobic until three decades ago when the first aerobic representative was identified [66]. In both aerobic and anaerobic taxa, bchl-*a* is the primary light-harvesting pigment and absorbs red light at 770 to 880 nm and blue light at ~385nm [67,68,69]. This provides a useful contrast to the chlorophyll-*a* present in cyanobacteria and algae, which absorbs at 430 nm and 665 nm (Figure 3).

The aerobic anoxygenic phototrophic (AAnP) bacteria are a diverse group of prokaryotes with respect to their functionality, physiology, and morphology [69]. These are obligate aerobes with unusually high concentrations of carotenoids, low cellular contents of bchl-*a* and a distinct lack of the light-harvesting complex II [38,39]. In the anaerobic phototrophic bacteria (AnPB), the *puf* operon coding for the bchl is repressed by both oxygen and high light [70]. However, in the AAnP, the expression of the *puf* operon is not limited by oxygen, but it is still repressed by strong light [37,71,72]. Despite these physiological differences, both AnPB and AAnPs have a similar photosynthetic apparatus and similar electron carriers and structural polypeptides [73]. The close correlation between AAnPs and oxygenic phototrophs in the euphotic zone may indicate that these cells contribute to a light-controlled component of the microbial redox cycle [38]. Several molecular studies based on the genes of the *puf* operon have been carried out in tropical, temperate and polar marine environments [74,75,76,77,78,79] and these organisms have been estimated to account for up to 10% of the energy production in the upper layers of the water column [38,80] in most temperate and tropical oceans. 

There is some evidence to suggest that AAnP bacteria are absent at high latitudes [81] and are genetically distinct from their freshwater counterparts [82,83]. However, positive *puf*M [64,73] clonal sequences were detected from extracted DNA and messenger RNA transcripts from the lower sections of Antarctic annual fast-ice as well as the underlying water column [84]. All clones grouped with the cultured α-Proteobacteria [39,74]. No β- and δ-Proteobacteria AAnPs were detected in the sea ice, matching the observations of Karr *et al.* [82] at Lake Fryxell. In fact, all the sea ice and seawater clones were likely α-Proteobacteria *Roseobacter*-clade affiliated [84], which could constitute ~20% of the Southern Ocean bacterial community [85,86], given that *Roseobacter denitrificans* is able to illicit specific defence systems against photo-oxidative stress [87]. More importantly, their presence in RNA extracts indicates that bacteria within the sea ice are actively expressing the gene for bchl synthesis. 

AAnPs may constitute only ~0.05% of the prokaryotic abundance in the Western Antarctic waters [80], however the ease with which AAnPs were found within sea ice suggests that their relative proportion may be higher in ice-associated microbial communities. Results obtained by quantitative PCR suggest that Bchl OTUs may comprise up to 10% of the sea ice bacterial community [88], although further work is clearly needed to ascertain the ecological significance of this metabolic pathway. 

### 2.3. Proteorhodopsin

The discovery of phototrophic energy generated via proteorhodopsin (PR) was a major finding in microbial ecology [89]. PRs are retinal binding bacterial integral membrane proteins that belong to the microbial rhodopsin super-family of proteins and function as light-driven proton pumps [89,90]. Unlike Bchl, PR cells do not generate cellular reducing power through NADPH, however ATP is produced upon light stimulation without the evolution of oxygen or fixation of inorganic carbon. Since the first reported PR sequence was obtained in 2000 [89], many other PR-bearing bacteria have been identified in environments ranging from freshwater lakes to the deep marine biosphere [91,92,93,94,95,96]. PR genes appear to be abundant in the genomes of oceanic bacteria [95], accounting for 13% of the prokaryotic community in the Mediterranean Sea and Red Sea and 70% in the Sargasso Sea [94,95,97,98]. Importantly, *in vitro* studies have demonstrated proton pumping and increased growth rates of PR-bearing bacteria under illuminated conditions [99,100,101,102]. Recently, PR in *Candidatus* Pelagibacter ubique was reported to play a critical role in a cellular response that maintains cell function during periods of carbon starvation [102]. These observations suggest that harvesting light energy via PR may be important in marine environments [103], but again the ecological significance of this metabolic pathway is currently unknown.

Sea ice bacteria that express the PR gene were described for the first time in 2010 [104]. PR-bearing representatives from the classes α-Proteobacteria, γ-Proteobacteria and Flavobacteria were present throughout the fast-ice in the Ross Sea region of Antarctica. Complementary DNA (cDNA) generated from RNA samples suggested that PR bacteria were metabolically active at the time of sampling. The bulk of the positive cDNA samples were collected from the middle and bottom part of the ice matrix, which possibly indicates that PR bacteria favor the lower light intensity and relatively stable temperatures found in the bottom half of the ice. Essentially, as light penetrates deeper into the ice matrix, the more energetic blue light predominates [105]. A stratified distribution of different forms of PR-bacteria in marine waters has been observed previously [106,107], and this has been attributed to a single-residue switch mechanism whereby the presence of leucine or glutamine at amino acid position 105 determines whether the protein absorbs in the green or blue wavelength [106]. Koh *et al.* [104] found both blue-absorbing (BPR) and green-absorbing (GPR) forms, but BPR were found primarily in the middle of the ice where red and green wavelengths of the solar spectrum are relatively low [105]. Conversely, GPR appear to be distributed throughout the ice, but their highest concentrations were at the ice/water interface [104], where, due to the presence of eukaryotic chl-*a*, the only available light is green. 

## 3. Future Research and the Significance of Light-Harvesting Pigments for Antarctic Sea Ice

Research-to-date has confirmed that some of the bacteria present in Antarctic sea ice are capable of phototrophic metabolism, most likely as a supplement to an otherwise heterotrophic lifestyle. A mechanistic understanding of the diversity, ecophysiology, and functionality of marine photoheterotrophs is therefore a worthy goal, but one that is extremely challenging [108].

Logistic and weather constraints are the primary reasons that polar studies are conducted during the summer months. As a result, insight into ice-associated light-harvesting bacteria has thus far come from cores extracted during the austral summer. In the future, it will be particularly important to contrast the light-driven energy flux with the metabolic processes and activity level that take place during the dark polar winter. Only a handful of over-winter studies have been reported in the more accessible Arctic [78,109,110], however microorganisms are more active during summer months compared with winter.

Next generation pyrosequencing [111,112], microfluidics [113] and microarray analysis [114,115,116] are rapidly changing the way microbial communities are studied. These high-throughput methods could be employed to elucidate more phototrophic bacteria from Antarctic sea ice and accurately determine their *in situ* distribution and abundance. Techniques such as catalyzed reporter deposition (CARD)-FISH [117] and quantitative PCR [80,118] will enable functional gene expression to be quantified at the single-cell level of resolution. In addition, metatranscriptomics and proteomics would provide a valuable tool with which to link *in situ* expression dynamics with environmental stress [99,119,120]. Coupled with the chromatin immune-precipitation (ChIP) procedure, it is now possible to characterize both the genome-wide location and function of novel energy-binding proteins [120,121,122].

## 4. Concluding Remarks

Sea ice represents one of the most ephemeral habitats on Earth and the ice-associated microbial communities are integral to the energy base of the Southern Ocean ecosystem. The specific physiological roles and adaptive strategies of phototrophic bacteria within this ecosystem have yet to be elucidated; however, the future looks promising given the expanding range of technologies that may be used to explore the bioenergetics of light-harvesting pathways. There is also a growing need to quantify the resilience of sea ice microbes to increased environmental stress and to provide a real-time biological response to climate change. Considering the variety of genetic, physiological and environmental contexts in which light-harvesting bacteria are found, the diversity observed to date may reflect only a subset of the organisms present and more light-dependent adaptive strategies are likely to exist in the microbial world. The combined sequencing of cultivated and uncultivated organisms will undoubtedly reveal more microbial groups with known, or even novel, photosynthetic abilities.
